# Evaluation of magnetic resonance imaging derived synthetic computed tomography for proton therapy planning in prostate cancer

**DOI:** 10.1016/j.phro.2024.100625

**Published:** 2024-08-12

**Authors:** Kajsa M.L. Fridström, René M. Winter, Natalie Hornik, Sigrun S. Almberg, Signe Danielsen, Kathrine R. Redalen

**Affiliations:** a Department of Physics, Norwegian University of Science and Technology, Trondheim, Norway; b Cancer Clinic, St. Olav Hospital, Trondheim, Norway; c Eberhard Karls University of Tübingen, Tübingen, Germany

**Keywords:** Synthetic CT, Radiotherapy planning, Prostate cancer, Proton therapy, MRI-only

## Abstract

**Background and purpose:**

Magnetic resonance imaging (MRI)-only workflow is used in photon radiotherapy (RT) today, but not yet for protons. To bring MRI-only proton RT into clinical use, proton dose calculation on MRI-derived synthetic CT (sCT) must be validated. We evaluated proton dose calculation accuracy of prostate cancer proton plans using a commercially available sCT generator already validated for photon planning.

**Materials and methods:**

The retrospective planning study included 10 prostate cancer patients who underwent MRI and planning CT (pCT) before RT. sCT were generated from the MRI with MRI Planner v2.3, and compared to pCT using structural mean absolute error (MAE). The pCT was used to create one-arc volumetric modulated arc therapy (VMAT) photon plan and two-field intensity modulated proton therapy (IMPT) proton plan. Each plan was recalculated on the sCT and compared to pCT doses. Dose volume histogram parameters, gamma analyses and range differences were evaluated.

**Results:**

Median MAE for the body contour was 71 HU. Dose differences between pCT and sCT were small and similar for VMAT and IMPT plans. Median (range) gamma pass rates were lower for IMPT plans with 95.8 (89.3–98.7) % compared to VMAT plans with 99.4 (91.2–99.6) %. The proton range difference was 1.0 (interquartile range –0.1 – 0.2) mm deeper for sCT compared to the reference.

**Conclusion:**

MRI-only IMPT planning for prostate cancer seems feasible in a clinical setting for the evaluated beam arrangement and sCT generator. More patients and evaluation of other beam arrangements are needed for a more general conclusion.

## Highlights

• Magnetic resonance imaging (MRI)-only is feasible for prostate cancer proton therapy.
• MRI-derived synthetic computed tomography (sCT) was comparable to planning CT (pCT).
• Dose difference between pCT and sCT was small and similar for photon and proton plans.
• Gamma pass rates were lower for proton plans than for photon plans.
• Analyzed Bragg peaks on sCT was 1 mm deeper than for pCT.

## Graphical abstract

**Figure:**
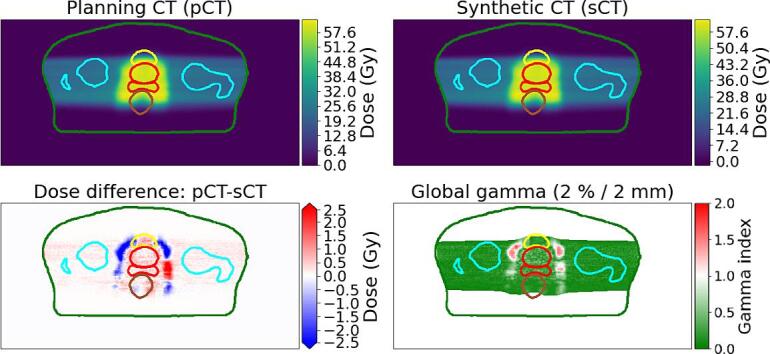

## 1 Introduction

Current guidelines recommend that magnetic resonance imaging (MRI) is used for target delineation in radiotherapy (RT) for prostate cancer [1]. The superior soft tissue contrast of T2-weighted MRI compared to CT results in reduced interobserver variability and improved accuracy in target delineation [2], [3] and is therefore recommended in guidelines [1]. Dose calculations for RT plans are commonly based on the relation between the CT Hounsfield Unit (HU) and electron density for photon RT [4] and mass density or stopping power ratio for proton RT [5], which makes the CT essential in the RT workflow. Transferring delineated structures from MRI to CT for dose calculation requires accurate image registration between the two modalities. This introduces the possibility of registration errors that can reduce the overall treatment accuracy [4], [6].

By utilizing an MRI-only workflow, registration errors are removed, and the total systematic spatial uncertainty is reduced. Since MRI lacks HU-information, MRI needs to be converted to so-called synthetic CT (sCT) that can provide the information needed for dose calculations [4]. This is a hot topic that engages many in the research community [7], [8] and several methods to generate sCT for photon RT have been published [9], [10], including in-house solutions [11], [12] and commercially available software [13], [14], [15], [16], [17].

Prostate cancer proton RT has shown advantages over photon RT in terms of reduced dose to normal tissues [18]. The integrated approach of the high soft tissue contrast from MRI and the favorable dose distribution of proton therapy is attractive. Other areas of research where MRI is integrated into the RT workflow includes developing MR-integrated proton RT systems to enable MRI-guided proton therapy, like what is already available for photon RT. However, several technical difficulties must be overcome [5], [10], [19], [20]. For online plan adaptation, MR images either need to be registered to a planning CT (pCT) or a sCT is required for dose calculation [10]. Patients with considerable tumor motion or anatomical variation, or patients where dose escalation with photons is currently unsafe due to nearby critical structures, are attractive for MRI-only proton RT [19].

Before clinical implementation of MRI-only proton RT, dose calculation accuracy on sCT must be ensured similarly as for photon RT [13], in addition to proton specific parameters. Some studies have evaluated proton dose calculation on sCT, but due to the novelty, knowledge on feasibility is not well established.

Recognizing that sCT is already validated for photon RT, our aim was to evaluate proton dose calculation accuracy of sCT for prostate cancer patients using a commercial sCT software validated for photons. Our investigation was based on MRI and CT acquired in RT position of 10 prostate cancer patients. Photon and proton RT plans were created based on the pCT and recalculated on the sCT for comparison. Since photon RT with MRI-only workflow is well established, the accuracy for dose calculation for proton RT plans on sCT was benchmarked against the calculations for the photon RT plan before dose differences were evaluated.

## 2 Materials and methods

### 2.1 Patients and image acquisition

Ten high risk prostate cancer patients, median age 72 (range 57–82) years and median body mass index 25 (range 22–31) kg/m^2^, referred to RT were included in an observational imaging study between September 2021 and June 2023 (NCT04790968). Patients with metal implants near the relevant region were excluded. The study was performed in accordance with the Helsinki Declaration, and all patients gave written informed consent. The study was approved by the Institutional Review Board and the Regional Committee for Medical and Health Research Ethics in Central Norway. The MRI and CT acquisition procedures are described in Supplementary Material A.

### 2.2 Synthetic CT generation and treatment planning

For all patients, one sCT was generated with MRI Planner v2.3 (Spectronic Medical AB, Helsingborg, Sweden) based on the large field of view T2-weighted MRI. MRI Planner uses a deep convolutional neural network based on the transform function estimation algorithm to automatically generate an sCT, retaining the same resolution as the original MRI [21]. Treatment planning procedures are described in Supplementary Material A.

### 2.3 Evaluation of geometrical variations and dose calculation accuracy

pCT and sCT with associated structure files and treatment plans were exported in DICOM format and analyzed using Python 3.9. First, mean absolute error (MAE) in CT image intensity was calculated for all N contour voxels of a structure as in [22], [23]:

(1) $$\mathrm{MAE}=\frac{\sum_{i=1}^{N}\left|pCT\left(i\right)-sCT\left(i\right)\right|}{N}$$

The calculation was performed for the body contour and femoral heads. Voxels outside the intersection of pCT and sCT contours of a structure were disregarded. Secondly, differences in dose volume histograms (DVH) between pCT and sCT were analyzed for both VMAT and IMPT plans, comparing both the mean DVH and the difference (pCT-sCT per prescribed dose) for selected DVH parameters. For OARs, near maximum dose, represented by the dose received by 2 % of the volume (D2), and median dose (D50) were examined. For all CTVs, D2, D50 and the minimum dose (D99.5) were evaluated. Since several dose levels are relevant for rectum toxicity, the relative volumes receiving 5 Gy (V5), 20 Gy (V20), 47 Gy (V47), 53 Gy (V53) and 56 Gy (V56) were analyzed. The volume receiving 59 Gy (V59) was analyzed for the anal canal, whereas the volumes receiving 58 Gy (V58) and 62 Gy (V62) were analyzed for the bladder. Statistical analysis of differences between pCT and sCT parameters for VMAT and IMPT, was assessed with the Wilcoxon signed rank test, using p < 0.05. The overall agreement, also outside the delineated volumes, was evaluated using global gamma index analyses [24] calculated with Python package PyMedPhys v.0.39.3 [25]. Analyses were performed for 3 %/3mm and 2 %/2mm with a dose cutoff at 10 % to assess overall dose deviation, and at 90 % to assess the prescribed dose delivered to the target areas. Only points present in both the pCT and sCT were analyzed. To achieve the same image resolution for gamma analysis, the sCT was resampled to the pCT using 3D Slicer v5.2.1 and the BRAINS Resample Image module with a bspline interpolation mode [26]. Differences in Bragg peak positions, i.e. range differences, between pCT and sCT, were evaluated for a subset of spots in both proton beams. The spots were associated with two energy-layers, representing two depths, one in the center and one in the distal part of the CTV. The evaluation was performed by manually measuring the difference between pCT and sCT Bragg peak positions in Plan Evaluation mode in RayStation.

## 3 Results

The median (interquartile range) MAE over all patients were 71 (44–99) HU for the body contour and 126 (92–145) HU for the femoral heads.

**Fig. 1:**
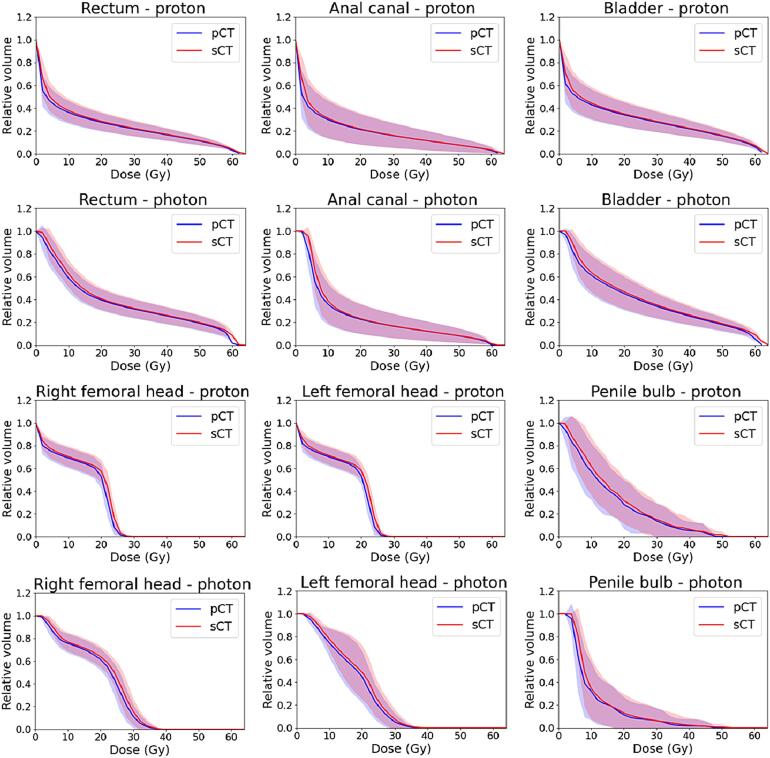
Organs at risk (OAR) mean (solid line) and standard deviation (shaded area) DVHs for proton plans (first and third row) and photon plans (second and forth row) for 10 prostate cancer patients. The blue curve is the DVH based on the conventional planning CT (pCT) and the red curve from the MRI-derived synthetic CT (sCT). (For interpretation of the references to colour in this figure legend, the reader is referred to the web version of this article.)

**Fig. 2:**
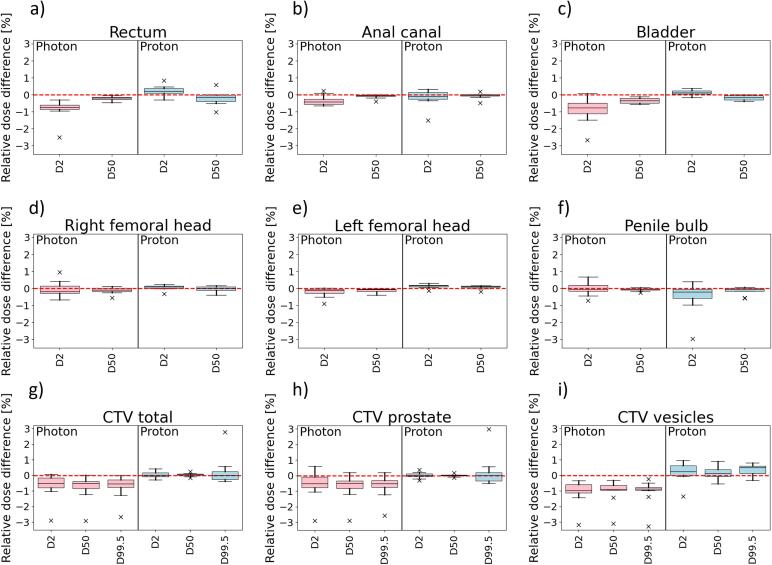
Dose difference (planning CT (pCT) – synthetic CT (sCT)) relative prescribed dose for DVH-parameters (n = 10) between treatment plans created using conventional pCT, recalculated on MRI-based sCT for photon and proton plans. The differences were statistically significant mainly for maximum dose (D2) for OARs. The 25th and 75th percentiles are presented by bottom/top of the boxes. The median is presented by the line inside each box.

**Fig. 3:**
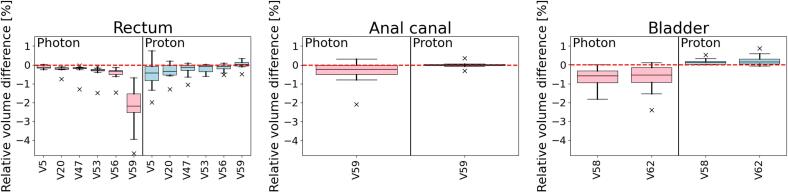
Volume difference (planning CT (pCT) – synthetic CT (sCT)) relative structure volume for DVH-parameters (n = 10) between treatment plans created using conventional pCT, recalculated on MRI-based sCT for photon and proton plans. The 25th and 75th percentiles are presented by bottom/top of the boxes. The median is presented by the line inside each box.

The mean VMAT and IMPT DVHs for OARs of all 10 patients are presented in Fig. 1. There were no significant differences between pCT and sCT across the whole DVH for neither VMAT nor IMPT plans. Individual DVHs for each patient can be seen in Supplementary Fig. S1. The total mean dose difference relative to the prescribed dose and the corresponding standard deviation (SD) for the combined CTV for all patients was 0.1 (SD = 0.1) % for IMPT and −0.8 (SD = 0.8) % for VMAT plans. The mean dose deviations were more similar for the OARs, where the bladder had the largest deviation for VMAT (mean = −0.4 %, SD = 0.2 %) and rectum had the largest deviation for IMPT (mean = -0.3 %, SD = 0.4 %). Boxplots of dose and volume differences are presented in Fig. 2, Fig. 3. When analyzing differences between D50 for VMAT compared to IMPT plans, a significant difference was found at D50 for left femoral head (p = 0.002). D2 deviated more and a significant difference was found for rectum (p < 0.001), bladder (p = 0.001) and left femoral head (p = 0.001). For the CTV, the difference was significant for D2 (p = 0.008), D50 (p = 0.001) and D99 (p = 0.006).

**Fig. 4:**
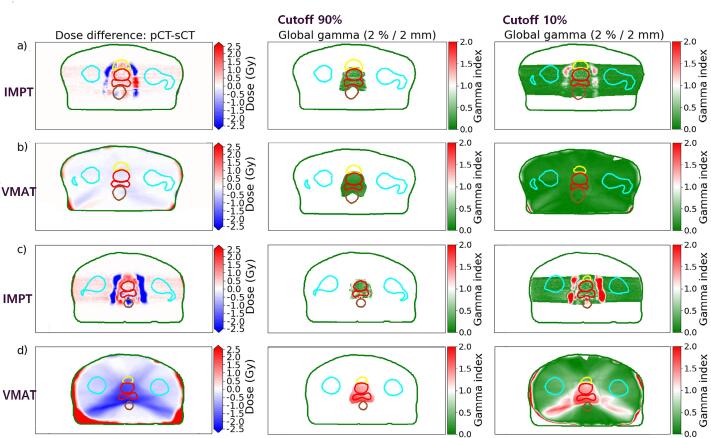
Representative example of a global gamma analysis for 2 %/2 mm for the intensity modulated proton therapy (IMPT) and volumetric modulated arc therapy (VMAT) plans of a patient with good registration (a), here represented by patient 2, and a patient with a bad registration (b), here represented by patient 8. Differences (planning CT – synthetic CT) in dose distribution are presented in the left column where red areas represent higher doses for the planning CT than the synthetic CT and blue areas represent lower doses. Gamma index for dose cutoff 90 % (middle column) and 10 % (right column) are presented with areas passing in green and not passing in red. Delineated structures are overlayed to better visualize location. Depicted is external contour (dark green), right and left femoral head (light blue), bladder (yellow), rectum (brown) and the combined CTV for seminal vesicles and prostate (red). (For interpretation of the references to colour in this figure legend, the reader is referred to the web version of this article.)

Global and local gamma analyses for 2 %/2mm are presented in Table 1 and for 3 %/3mm in Supplementary Table S1. The median (range) local gamma pass rate for dose cutoff 90 % was 97.3 (86.6–99.9) % for IMPT plans, and 100 (9.7–100) % for VMAT plans. For a dose cutoff of 10 %, the 2 %/2 mm gamma pass rate was 94.9 (85.1–98.1) % for IMPT plans and 97.7 (78.7–98.5) % for VMAT plans). Two representative patients, one with good and one with poor image registration, are presented in Fig. 4, illustrating the gamma index results for the 10 % and 90 % cutoff, 2 %/2mm global gamma analysis, for IMPT and VMAT plans. The largest absolute dose differences for IMPT between the two (Fig. 4, left column) were observed just outside the target for both patients. For VMAT, the largest discrepancies were evenly distributed and related to contour differences. The gamma index distributions are presented in Fig. 4, middle and right column.

**Table 1:** Global gamma index pass rates comparing dose calculated on the synthetic CT (sCT) compared to reference dose calculated on the planning CT (pCT) for proton intensity modulated proton therapy (IMPT) and photon volumetric modulated arc therapy (VMAT) plans for 2 %/2mm local and global analysis, lower dose cutoff at 10 % and 90 %. Percentages represent number of approved (gamma index < 1) voxels out of all voxels with a dose higher than the applied dose cutoff.

	10 % cutoff				90 % cutoff			
	Global gamma (%)		Local gamma (%)		Global gamma (%)		Local gamma (%)	
Patient	VMAT	IMPT	VMAT	IMPT	VMAT	IMPT	VMAT	IMPT
1	99.5	97.4	98.3	96.9	100	98.0	100.0	97.8
2	99.6	98.7	98.5	98.1	100	99.9	100.0	99.9
3	99.5	93.5	97.2	91.9	100	92.4	100.0	91.5
4	99.5	98.0	98.0	97.3	100	99.7	100.0	99.6
5	99.2	95.8	97.0	95.0	100	97.8	100.0	97.5
6	97.9	89.3	90.3	85.1	95.0	88.0	94.1	86.6
7	99.4	95.8	97.4	94.8	99.9	97.7	99.8	97.2
8	91.2	92.9	78.7	91.7	12.3	95.3	9.7	94.6
9	99.4	95.2	98.0	94.0	100	96.3	100.0	95.6
10	99.5	96.3	98.5	95.5	100	97.8	100.0	97.4
Median	99.4	95.8	97.7	94.9	100	97.7	100.0	97.3
Range	91.2–99.6	89.3–98.7	78.7–98.5	85.1–98.1	12.3–100	88.0–99.9	9.7–100	86.6–99.9

**Fig. 5:**
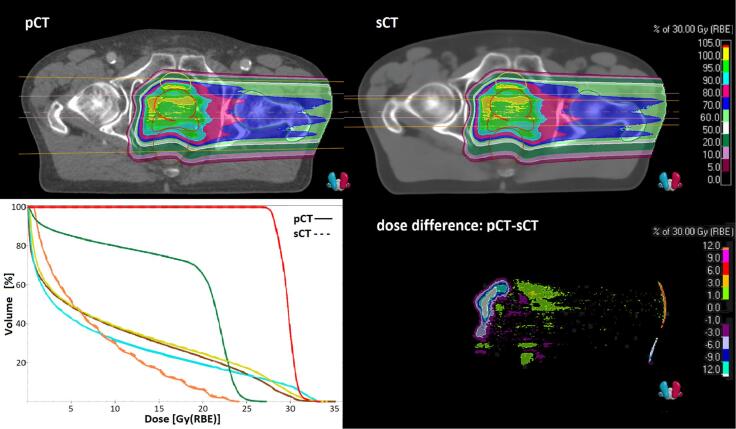
Representation of one of the proton fields (gantry 90°) that compose the proton plan for patient 2. Top row shows the dose contribution for the field for planning CT (pCT) (left) and synthetic CT (sCT) (right). The dose difference (pCT-sCT) (bottom row, right) displays the actual dose difference in percent. There is a slightly higher dose for the pCT before the target, and a lower dose behind. DVH (bottom row, left) shows the pCT dose (line) and the sCT (dotted line).

The median (interquartile range) spot displacement was 1 (−1–2) mm in the middle of the CTV and 1 (−1–3) mm at the distal edge. When considering the two fields separately, the displacement of the left lateral field was 2 (0–3) mm in the center of the CTV and 2 (1–4) mm at the distal edge. The right lateral field had a displacement of −1 (−2–1) mm in the center and 0 (−2–2) mm at the distal edge. Positive values represent that the points for the pCT are deeper than for the sCT in the field direction. An example of a dose distribution and associated DVH is presented in Fig. 5.

## 4 Discussion

We investigated the dose calculation accuracy of MRI-derived sCT for MRI-only IMPT planning for 10 prostate cancer patients. The study was performed using a commercial sCT software developed for photon RT, and the doses were compared to conventional pCT as well as to the corresponding accuracy for photon dose calculation, since the workflow for photon RT already is established and verified for clinical use. The dose difference of sCT relative to pCT was small and similar for both IMPT and VMAT plans. The main discrepancies were seen in gamma analyses for areas outside the delineated target volumes. We found that the calculated dose on sCT and pCT (Fig. 1, Fig. 2, Fig. 3) were similar for both OARs and CTV. For both photon and proton dose calculation, the relative dose difference for selected dose parameters for CTV, bladder, rectum, anal canal and femoral heads were mostly within ±1 % (Fig. 2), which is in accordance with Koivula et al [27], although they did not report corresponding results for photons. Thus, for current field setup and our selection of patients, we do not expect the introduction of sCT in the treatment planning workflow to cause any systematic over- or under dosage to the volumes of interest. Outliers for the dose deviation were up to 3 % and most common for CTVs of VMAT plans, and more frequent for maximum doses than median doses. It seems like outliers could be explained by visually identified anatomic differences due to imperfect image registration. Inaccuracies in bone position were most important for IMPT plans, and deviation in the external contour of most importance for VMAT plans. Deviations in the external contour affected dose difference both for VMAT and IMPT plans, but the difference was most evident for VMAT plans since deviations in all directions accumulate throughout the arc, unlike for the IMPT plan that only have two incident field angles. This is affecting the CTV dose for the VMAT plans and results in systematic overestimations of the recalculated sCT doses. These latter errors are not relevant for the comparison of dose calculation performance between pCT and sCT but are relevant in a clinical treatment setting.

MAE for HU-differences has previously been reported by other studies of sCT in prostate cancer [22], [23]. Kim et al. [22] used an in-house voxel-based weighted summation approach to generate sCT from MRI and found a mean MAE of 74 HU for the body contour, in accordance with 71 HU found in this study. Siversson et al. [23] on the other hand, found a lower MAE of 37 HU for the body contour. The authors used an earlier version (MRI Planner v1.1) of the sCT software than us however, they used a deformable registration approach in contrast to rigid registration approaches used in [22] and this study, which might explain a lower MAE. Accurate registration is important to help mitigate unintentional effects from geometrical variations between the CT and MRI, which can arise e.g., from patient repositioning between imaging examinations or differences in bladder or rectal filling. Such effects should be minimized to best possibly assess the true performance of the sCT algorithm. In our study, image registration between CT and MRI was done based on bony anatomy to minimize the influence of errors originating from anatomical changes such as different rectum and bladder filling, as well as positional uncertainties. HU errors related to inaccurate representation of bone and air will affect the dose calculation and might introduce errors for sCT in proton RT [28]. Since protons are sensitive to different uncertainties, robust optimization must be applied for proton RT [29]. Deformable image registration was not done due to the risk of introducing additional errors [27], [30], [31], [32].

Dependency on dose uncertainty for proton fields with incident angle has been reported. For instance, the differences in dose for anterior compared to lateral proton fields for prostate cancer, is believed to be reduced because of the placement of the femoral heads relative to the prostate [33]. Others have also suggested that the beam setup used here might be the worst possible since all other beam arrangements result in a smaller path through bone, and hence a reduced error in proton dose calculation for an MRI-only workflow [30]. However, this field setup is the most used for proton RT of the prostate today.

Global gamma analysis was performed to provide information about the accuracy of dose calculations outside of delineated structures. Our median global gamma pass rates for 2 %/2mm with a dose cutoff at 10 % were 95.8 % for IMPT and 99.4 % for VMAT (Table 1). The results of our global gamma analysis for prostate IMPT are consistent with similar analyses by others [27], [30], [34]. The global gamma analysis is known to be less sensitive to steep dose gradients and deviations in low dose areas compared to local gamma analyses [35], but the parameter was included to enable comparison with earlier relevant publications [27], [30], [34]. We also performed the analysis for local gamma (Table 1 and Supplementary Table S1). The largest impact of using local gamma was seen for VMAT plans at cutoff 10 % for patients with a large contour difference. Our results for the median 2 %/2mm global gamma analysis for IMPT (95.3 %, SD 2.8 %, Supplementary Table S1) showed slightly worse pass rates than previous studies by Koivula et al [27] and Maspero et al [30], who reported a mean pass rate of 98.6 % and 98.4 %, respectively. Both used a common body contour for pCT and sCT by setting a HU representing water or fat in areas with no tissue between external contours. This method has earlier been shown to improve results when analyzing photon MRI-only workflow [13]. This was not done in this study, instead we used the intersection of points between the body contours for the pCT and the sCT. This gives an estimate of the upper limit of the gamma pass rate. No alterations were performed for the different images, neither through changing contours nor by performing deformable registration. Núñez et al reported a 2 %/2 mm gamma pass rate of 95.1 (SD 6.8) % [34]. The points that fail for the gamma analysis of the IMPT plans are mainly in the area just outside the target volume for all patients (Fig. 4, middle and right column). This coincides with the end of range of the proton beam, visualized in Fig. 5 (lower right) [36]. This is to be expected since errors will accumulate along the beam path of proton beams and has also been observed and discussed by others [30], [36]. Global gamma analysis was also performed for dose cutoff 90 % to further assess the dose received by the target area (Table 1). The gamma pass rate for IMPT plans improved compared to the lower dose cutoff. VMAT pass rates also improved for all but two patients, with pass rates close to 100 %. The pass rate for the two remaining patients decreased. One of them had a decrease of 78.9 % for the 2 %/2mm when changing from dose cutoff 10 % to 90 %. This implies that IMPT plans seem to give a homogenous error distribution, while errors for VMAT plans can in some cases be concentrated to the actual target volume for large contour differences.

The range uncertainty of proton beam depends on many factors related to uncertainties in the assessment of the relative stopping power of all tissues, such as accuracy of the CT calibration curve mass density and relating stopping power to HU, how the dose calculation algorithm handles heterogeneities, as well as geometric and anatomic uncertainties [28], [29], [37], [38]. Our range uncertainty was found to be 1 mm in both the central and distal part of the CTV. Robust optimization has been shown to account for range uncertainties introduced by sCT dose calculations. Although uncertainties related to the introduction of sCT in the MRI-only workflow must be added to already existing uncertainties for proton RT [5], [10], [39], [40], the geometric uncertainties originating from CT to MRI registration are removed. This uncertainty has been estimated to be around 2 mm [6]. Possible improvements to further reduce the overall uncertainty are to create proton specific sCTs based on proton stopping power instead of electron stopping power [41], or to create sCT based on dual energy CT information known to be more accurate for proton dose calculations and enable reduction in range uncertainty compared to regular single CT calculations [42].

In summary, MRI-only proton therapy planning for prostate cancer is feasible when considering two lateral opposed fields and using a commercially available deep learning sCT generator. The differences observed, both between sCT and pCT and between IMPT and VMAT plans, are well below what is relevant in a clinical situation. However, care should be taken for other field arrangements and the impact on range uncertainties should be examined further.

## CRediT authorship contribution statement

**Kajsa M.L. Fridström:** Conceptualization, Methodology, Validation, Formal analysis, Investigation, Writing – original draft, Writing – review & editing, Project administration. **René M. Winter:** Methodology, Validation, Writing – review & editing. **Natalie Hornik:** Methodology, Formal analysis, Visualization, Writing – review & editing. **Sigrun S. Almberg:** Investigation, Conceptualization, Writing – review & editing. **Signe Danielsen:** Supervision, Conceptualization, Writing – review & editing. **Kathrine R. Redalen:** Supervision, Conceptualization, Writing – review & editing.

## Declaration of competing interest

The authors declare that they have no known competing financial interests or personal relationships that could have appeared to influence the work reported in this paper.
