# Supplementary material for: Evaluation of magnetic resonance imaging derived synthetic computed tomography for proton therapy planning in prostate cancer
Source: Phys Imaging Radiat Oncol. 2024 Aug 12;31:100625. doi: 10.1016/j.phro.2024.100625 (PMC11381754; doi:10.1016/j.phro.2024.100625)
Supplement: Supplementary Data 1 [file mmc1.pdf]

Supplementary Materials

Supplementary Material A

MRI and CT image acquisition

Study-specific MRI was acquired on a 3T PET/MRI system (Siemens Biograph mMR, Siemens Healthineers, Erlangen, Germany) after hormone therapy. The scanner was equipped with a flat tabletop (Medibord Limited, Nottingham, UK) and a pelvic coil holder (Medibord) with a body matrix coil. The MRI was acquired using a coil holder to not deform the outer contour of the patient. The patients were immobilized according to radiotherapy (RT) setup procedures during imaging. Routine bowel emptying preparations for MRI acquisitions were performed, in addition to emptying of the bladder 30 minutes prior to MRI examination, followed by drinking one glass of water.

The MRI protocol consisted of a high-resolution three-dimensional turbo spin echo T2-weighted sequence (SPACE) with a field of view (FOV) enclosing the whole patient contour to enable sCT generation (slice orientation: transversal, FOV: 400 mm, echo time: 89 ms, repetition time: 1200 ms, slice thickness: 1 mm, pixel size: 0.5x0.5 mm^2). All patients had gold fiducial markers implanted to simplify image registration between pCT and MRI based on prostate localization. The fiducial markers were localized with a T1-weighted sequence (slice orientation: transversal, FOV: 250 mm, echo time: 2.5 ms, repetition time: 5.1 ms, slice thickness: 2 mm, pixel size: 0.6x0.6 mm^2).

The planning CT (pCT) was acquired on Philips Brilliance Big Bore (n=6) or Siemens Somatom go.Open Pro system (n=4) approximately one hour after the MRI with the same patient setup and without any additional emptying or drinking. The CT protocol consisted of 2 mm slices and a tube voltage of 120 keV.

Treatment planning procedures

For treatment planning and evaluation, the MRI, pCT and sCT were imported into RayStation 12A treatment planning system (RaySearch Laboratories AB, Stockholm, Sweden). The MRI and pCT were rigidly co-registered based on fiducial markers before the radiation oncologist delineated the combined clinical target volume (CTV) containing the prostate volume and seminal vesicles on the MRI. In addition, rectum, anal canal, bladder, femoral heads and penile bulb were delineated as organs at risk (OAR). To avoid potential effects from air cavities in the rectum on dose calculations, the rectum volume was set to water for the pCT. As a default, sCT images were generated with the rectum volume set to water in MRI Planner. One photon volumetric modulated arc therapy (VMAT) and one intensity-modulated proton therapy (IMPT) treatment plan (3 Gy x 20 fractions) for the prostate and seminal vesicles were created on the pCT for each patient. The prescription was set to median dose to the combined CTV. The VMAT plan consisted of one 6 MV arc and was planned according to local clinical practice on an Elekta Versa HD with 5 mm multileaf collimator leaves. The IMPT plans were planned using two lateral opposed fields. The IMPT optimization was made robust for the maximum dose to the patient and the

minimum dose to the CTV. A position uncertainty of 5 mm in all directions and a systematic density uncertainty of +/- 3% was used as robust settings. Both VMAT and IMPT plans were calculated with a uniform dose grid resolution of 1 mm. All plans and volumes were copied from the pCT to the sCT using rigid registration based on bone structures before the dose was recalculated on the sCT for both VMAT and IMPT plans. All dose calculations in RayStation use the Monte Carlo (MC) algorithm for proton- and collapsed cone (CC) for photon calculations.

Supplementary Figure S1

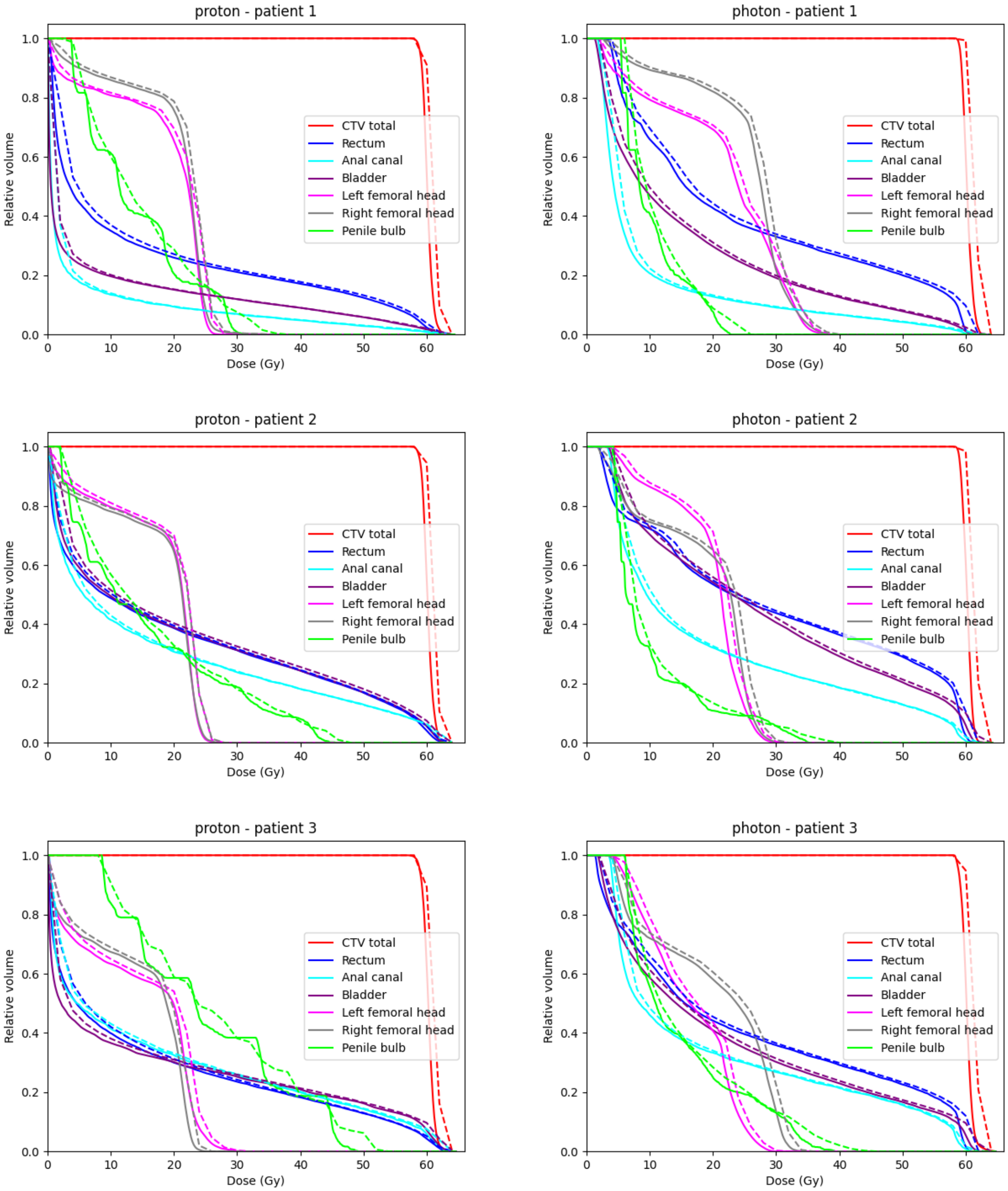

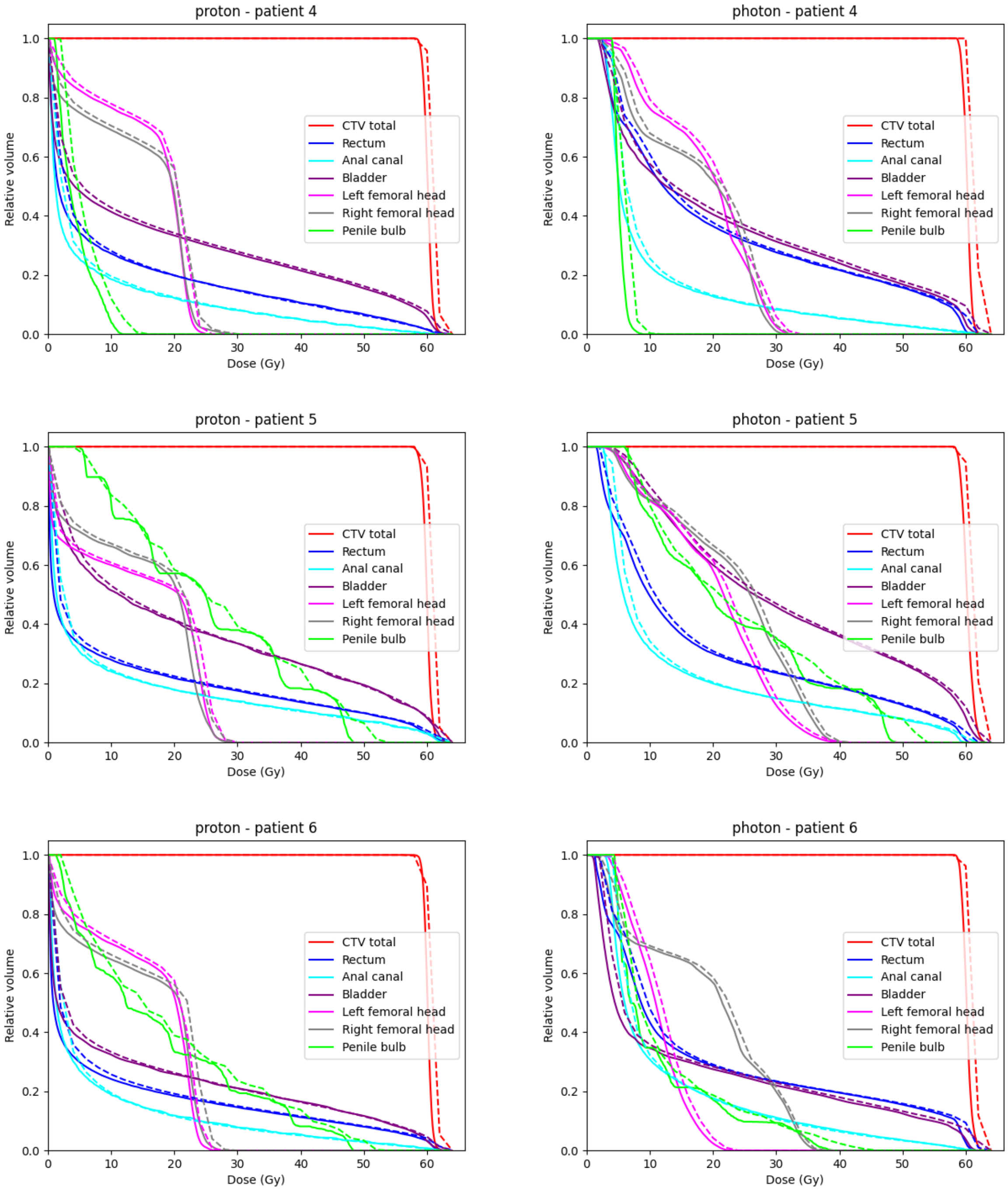

proton - patient 4
photon - patient 4
proton - patient 5
photon - patient 5
proton - patient 6
photon - patient 6
Relative volume
Dose (Gy)
0.0
0.2
0.4
0.6
0.8
1.0
0
10
20
30
40
50
60
CTV total
Rectum
Anal canal
Bladder
Left femoral head
Right femoral head
Penile bulb

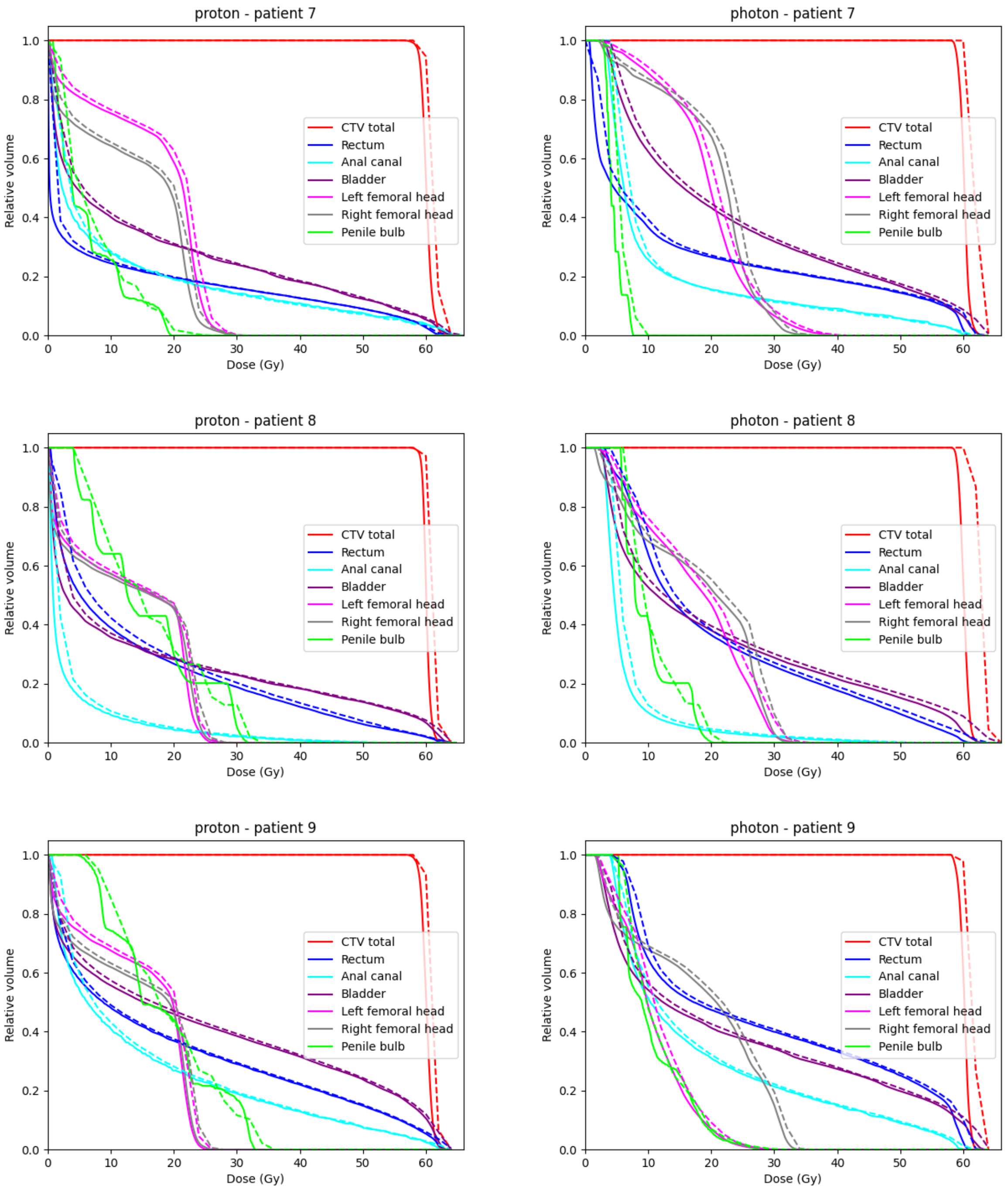
proton - patient 7
photon - patient 7
proton - patient 8
photon - patient 8
proton - patient 9
photon - patient 9
Relative volume
Dose (Gy)
0.0
0.2
0.4
0.6
0.8
1.0
0
10
20
30
40
50
60
CTV total
Rectum
Anal canal
Bladder
Left femoral head
Right femoral head
Penile bulb

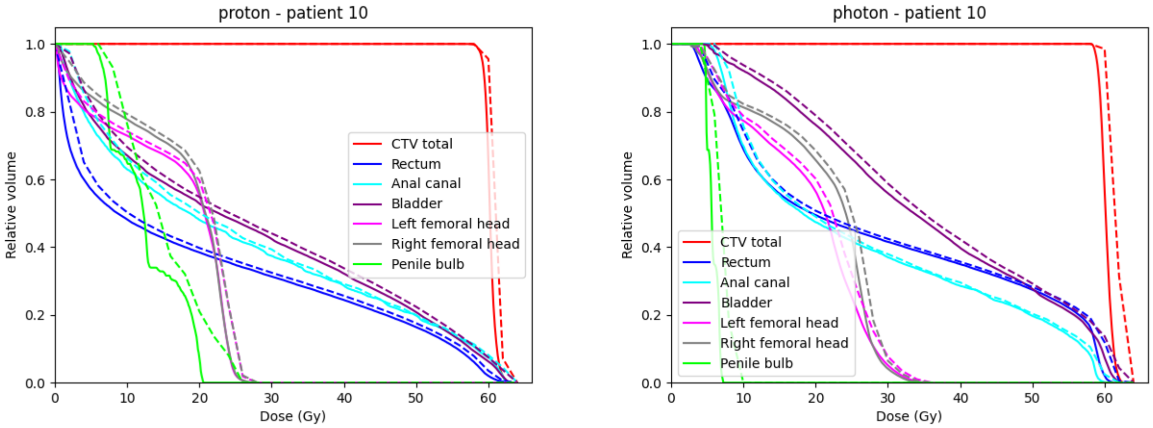

Supplementary Figure S1: Patient specific dose volume histograms (DVHs) for protons (left) and photons (right) where the dose for the synthetic CT (sCT) is displayed (dotted line) together with the dose for the planning CT (pCT) (solid line). Solid line represents the pCT and dotted line represents the sCT.

Supplementary Table S1

Global and local gamma index analysis for 2%/2mm and 3%/3mm and dose cutoff 10% (top) and 90% (bottom)

	LOWER DOSE CUTOFF 10%							
	proton				photon			
	global		local		global		local	
Patient	2%/2mm	3%/3mm	2%/2mm	3%/3mm	2%/2mm	3%/3mm	2%/2mm	3%/3mm
1	97.4	98.9	96.9	98.6	99.5	99.9	98.3	99.3
2	98.7	99.8	98.1	99.6	99.6	99.9	98.5	99.4
3	93.5	96.2	91.9	95.2	99.5	99.8	97.2	98.7
4	98.0	99.4	97.3	99.1	99.5	99.8	98.0	99.0
5	95.8	97.9	95.0	97.3	99.2	99.6	97.0	98.6
6	89.3	92.7	85.1	90.5	97.9	98.9	90.3	95.9
7	95.8	97.8	94.8	97.2	99.4	99.8	97.4	98.8
8	92.9	95.9	91.7	94.9	91.2	98.1	78.7	90.5
9	95.2	97.3	94.0	96.6	99.4	99.7	98.0	99.0
10	96.3	98.1	95.5	97.6	99.5	99.7	98.5	99.2
median	95.8	97.8	94.9	97.2	99.4	99.8	97.7	98.9
min	89.3	92.7	85.1	90.5	91.2	98.1	78.7	90.5
max	98.7	99.8	98.1	99.6	99.6	99.9	98.5	99.4
range	9.4	7.1	13.0	9.1	8.4	1.8	19.8	9.0
mean	95.3	97.4	94.0	96.7	98.5	99.5	95.2	97.8
sd (sample)	2.8	2.1	3.8	2.6	2.6	0.6	6.3	2.8

LOWER DOSE CUTOFF 90%

Patient	proton global 2%/2mm	proton global 3%/3mm	proton local 2%/2mm	proton local 3%/3mm	photon global 2%/2mm	photon global 3%/3mm	photon local 2%/2mm	photon local 3%/3mm
1	98.0	99.5	97.8	99.4	100.0	100.0	100.0	100.0
2	99.9	100.0	99.9	100.0	100.0	100.0	100.0	100.0
3	92.4	97.1	91.5	96.7	100.0	100.0	100.0	100.0
4	99.7	100.0	99.6	100.0	100.0	100.0	100.0	100.0
5	97.8	99.5	97.5	99.4	100.0	100.0	100.0	100.0
6	88.0	95.4	86.6	94.6	95.0	100.0	94.1	99.9
7	97.7	99.6	97.2	99.5	99.9	100.0	99.8	100.0
8	95.3	98.5	94.6	98.3	12.3	84.5	9.7	74.9
9	96.3	99.2	95.6	99.0	100.0	100.0	100.0	100.0
10	97.8	99.4	97.4	99.4	100.0	100.0	100.0	100.0
median	97.7	99.5	97.3	99.4	100.0	100.0	100.0	100.0
min	88.0	95.4	86.6	94.6	12.3	84.5	9.7	74.9
max	99.9	100.0	99.9	100.0	100.0	100.0	100.0	100.0
range	11.9	4.6	13.3	5.4	87.7	15.5	90.3	25.1
mean	96.3	98.8	95.8	98.6	90.7	98.5	90.4	97.5
sd (sample)	3.6	1.5	4.0	1.7	27.6	4.9	28.4	7.9
